# Cross-Reactive Immune Response of Bovine Coronavirus Spike Glycoprotein to SARS-CoV-2 Variants of Concern

**DOI:** 10.3390/ijms252111509

**Published:** 2024-10-26

**Authors:** Chiara Cossu, Valentina Franceschi, Antonino Di Lorenzo, Elisabetta Bolli, Sergio Minesso, Camilla Cotti, Laura Conti, Gaetano Donofrio

**Affiliations:** 1 Molecular Biotechnology Center “Guido Tarone”, Department of Molecular Biotechnology and Health Sciences, University of Turin, Piazza Nizza 44, 10126 Turino, Italy; chiara.cossu@unito.it (C.C.); antonino.dilorenzo@unito.it (A.D.L.); elisabetta.bolli@unito.it (E.B.); 2 Department of Veterinary Science, University of Parma, 43126 Parma, Italy; valentina.franceschi@unipr.it (V.F.); sergio.minesso@unipr.it (S.M.); camilla.cotti@studenti.unipr.it (C.C.)

**Keywords:** severe acute respiratory syndrome coronavirus 2 (SARS-CoV-2), bovine coronavirus, spike, immune cross-reaction, vaccination, bovine herpesvirus 4, COVID-19

## Abstract

The high variability observed in the clinical symptoms of severe acute respiratory syndrome coronavirus 2 (SARS-CoV-2) infections has been attributed to the presence, in a proportion of infection-naive subjects, of pre-existing cross-reactive immune responses. Here, we demonstrate that the bovine coronavirus spike protein (BoS) may represent a source of protective immunity to SARS-CoV-2. Indeed, vaccination of BALB/c mice with a Bovine herpesvirus 4 (BoHV-4)-based vector expressing BoS induced both cell-mediated and humoral immune responses that cross-react with SARS-CoV-2 spike protein. Although the spike-specific antibodies induced by BoS did not neutralize SARS-CoV-2, the T lymphocytes activated by BoS were able to induce cytotoxicity of cells expressing spike proteins derived from several SARS-CoV-2 variants. These results demonstrate that immunization with BoS may represent a source of cross-reactive immunity to SARS-CoV-2, and that these cross-reactive immune responses may exert protective functions. These results contribute to deciphering the mechanisms responsible for lack or mildness of symptoms observed in many individuals upon SARS-CoV-2 infection and may open new ways for the development of new vaccines for coronaviruses.

## 1. Introduction

The clinical manifestations of COVID-19, caused by severe acute respiratory syndrome coronavirus 2 (SARS-CoV-2), range from asymptomatic infection to respiratory failure. One of the reasons for this wide range of infection outcomes has been attributed to the presence, in some unexposed individuals, of cross-reactive immune responses that may influence susceptibility to infection and disease severity. The high homology of SARS-CoV-2 with other members of the coronaviruses (CoVs) family suggested that some of these viruses could be responsible for the induction of these cross-reactive immunity [1,2]. CoVs constitute the largest known family of RNA viruses, possessing a positive-sense, single-stranded RNA genome. Several CoVs infecting humans (HCoVs) have been identified and are classified into two groups: alpha-CoVs, which encompass HCoV-NL63 and HCoV-229E, and beta-CoVs, consisting of HCoV-HKU1, HCoV-OC43, MERS-CoV, SARS-CoV, and SARS-CoV-2 [3]. While MERS-CoV, SARS-CoV, and SARS-CoV-2 induce severe diseases with high morbidity and mortality rates, HCoV-OC43, HCoV-NL63, HCoV-229E, and HCoV-HKU1 are endemic viruses that typically induce mild upper respiratory tract infections [4]. Some studies described the presence of T cells and antibodies specific for other CoVs that cross-react with the SARS-CoV-2 spike (S) protein in SARS-CoV-2 unexposed donors [5,6,7]. Nevertheless, the precise origin and the subsequent function of pre-existing SARS-CoV-2 cross-reactive immunity remain to be elucidated. The characterization of cross-reactive immune responses to SARS-CoV-2 might improve our comprehension of the heterogeneity in COVID-19 patients’ outcomes and in the responses to SARS-CoV-2 vaccines.

Besides human CoVs, another source of cross-reactive immunization could be represented by bovine coronavirus (BCoV), which causes respiratory infections, winter dysentery, and (neonatal) calf diarrhea in cattle [8]. BCoV is widely diffused in livestock farms around the world and causes serious economic losses in the global cattle industry [9]. Notably, BCoV interspecies transmission can generate recombinant strains potentially able to escape immune response and to spread to other species including humans, as previously reported [10,11], potentially causing a zoonotic infection [4,12]. BCoV displays high similarity with HCoV-OC43 and shares with it the same receptor [13]. Indeed, both HCoV-OC43 and the BCoV spike glycoprotein (BoS) are responsible for the virus’s attachment to a modified sialic acid residue (9-O-Acetyl-N-acetyl-neuraminic) that acts as a receptor on the host cell and induces the fusion of the virus envelope with the cell membrane, leading to the formation of syncytia [14]. More recently, class I human leucocyte antigen (HLA) has been identified as an entry receptor for BCoV on human cells, suggesting that BCoV may infect humans, leading to the generation of an immune response [15]. Of note, BCoV shares some epitopes with SARS-CoV-2, which suggests the possible existence of cross-immunity between the two viruses [16,17].

In order to verify this hypothesis, we generated a viral vector encoding for BoS, based on the Bovine herpesvirus 4 (BoHV-4). BoHV-4 is a double stranded DNA γ-herpesvirus isolated from cattle that can be successfully used for vaccine therapy, since it shows several characteristics that explain its safeness as viral vector. Indeed, BoHV-4 is easy to be manipulated as a bacterial artificial chromosome (BAC) and can be leveraged for sustained transgene expression, thereby eliciting a strong host immune response [18,19].

## 2. Results

### 2.1. Generation and Characterization of a Viral Vector Encoding for BoS

**Figure 1:**
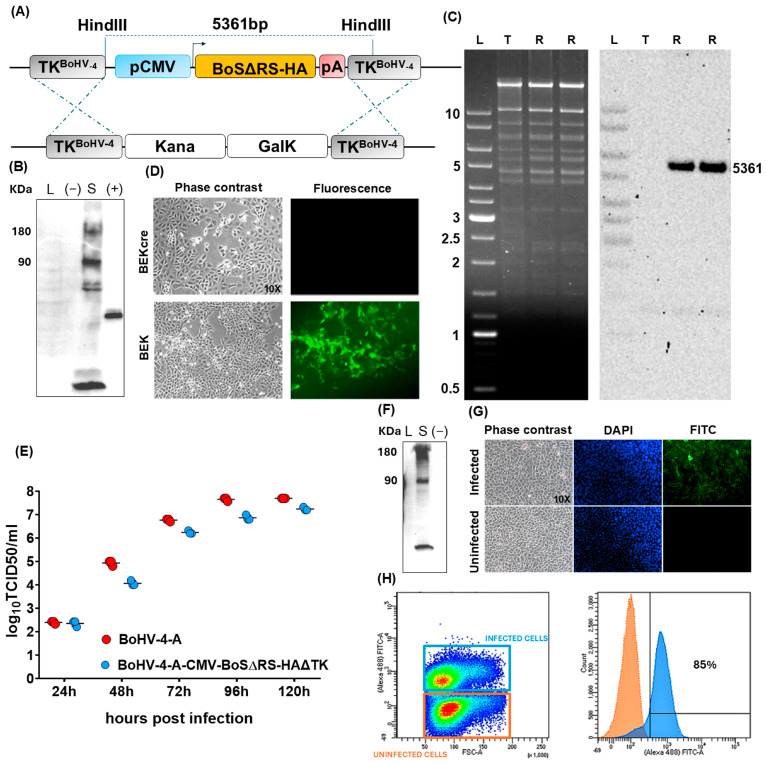
Design and production of BoHV-4-A-BoSΔRS-HA-ΔTK viral vector and its characterization. (**A**) Illustration (not to scale) describing the homologous recombination induced by heating in SW102 cells containing pBAC-BoHV-4-A-TK-KanaGalK-TK, where the Kana/GalK cassette was substituted with the CMV-S-BoSΔRS-HA expression cassette flanked by BoHV-4 TK sequences, cloned in the pINT2 (pINT2-(TK-CMV-BoSΔRS-HA-TK) shuttle plasmid. (**B**) Western immunoblotting of HEK293T cells transfected with the pINT2-(TK-CMV-BoSΔRS-HA-TK) vector (S; 40 µg of protein extracts), the pEGFP-C1 plasmid ((−); 40 µg of protein extracts) employed as a negative control, and HA tagged unrelated protein used as a positive control (+). (**C**) Representative agar gel electrophoresis of HindIII restriction enzyme analysis of 2-deoxy-galactose-resistant colonies (pBoHV-4-A-BoSΔRS-HAΔTK; R (Retargeted), compared with the parental pBoHV-4-A-Kana/GalKΔTK (T (Targeted)). The un-retargeted pBAC-BoHV-4-A-TK-KanaGalK-TK control 2650 bp band was substituted by a 5361 bp band in BoHV-4-A-S-ΔRS-HA-ΔTK (BoHV-4-A-spike), which, however, is not evident since it is overlaid on other bands of similar dimension. The right panel shows the Southern blotting, where a CMV-specific DNA probe confirmed the retargeting of interest. (**D**) Representative images of phase contrast and fluorescent fields of plaques formed by viable reconstituted recombinant BoHV-4-A-BoSΔRS-HAΔTK after the corresponding BAC DNA was electroporated into BEK cells or in BEK cells expressing cre recombinase (Magnification, ×10). (**E**) Replication kinetics of BoHV-4-A-BoSΔRS-HA-ΔTK growth on BEK cells, compared with those of the parental BoHV-4-A isolate. The graph shows means ± SEM of three independent experiments. *p* > 0.05 for all time points (Student’s *t* test). (**F**) Western immunoblotting of cells infected with BoHV-4-A-BoSΔRS-HAΔTK (S), the negative control (−) with protein extract from BoHV-4-A infected cells. The lanes were loaded with 20 μg of protein extract. (**G**) Immunofluorescent staining of BoHV-4-A-BoSΔRS-HAΔTK infected BEK cells and the uninfected control. Since BoS was HA tagged, an anti-HA monoclonal antibody was used. Counterstaining was performed with DAPI. (**H**) Cell surface expression of BoS in BEK cells infected with BoHV-4-A-BoSΔRS-HA-ΔTK at a multiplicity of infection of 0.1. was analyzed by flow cytometry 48 h post infection. The overlaid density and histogram plots differentiate the infected cells expressing BoS (blue square and histogram) from the uninfected control (orange square and histogram), showing that ~85% of cells were positive for BoS.

A recombinant BoHV-4, BoHV-4-CMV-BoSΔRS-HA-ΔTK, delivering the expression cassette for BCoV *spike* glycoprotein (BoS; https://www.uniprot.org/uniprotkb/A0A679DWT9/entry, accessed on 4 March 2023) was generated. First, the ORF encoding for BoS was designed in silico, substituting the last 57 bp, coding for a potential endoplasmic reticulum retrieval signal, with 27 bp coding for the hemagglutinin (HA) tag and human codon usage adapted with the Jcat codon usage adaptation software (http://www.prodoric.de/JCat, accessed on 4 March 2023) [2].These modifications, previously tested for other spike proteins coming from different coronaviruses, allowed a better cell surface expression and detection [20]. The BoSΔRS-HA sequence was chemically synthesized and inserted in a BoHV-4 shuttle vector, pINT2 [18], under the transcriptional control of the CMV promoter, the bovine growth hormone polyadenylation signal and flanked by two homologous regions of BoHV-4 TK gene to generate pINT2-(TK-CMV-BoSΔRS-HA-TK) (Figure 1A). BoSΔRS-HA expression was assessed by pINT2-(TK-CMV-BoSΔRS-HA-TK) transient transfection in HEK293T cells (Figure 1B). Then, recombinant pBAC-BoHV-4-A-BoSΔRS-HA-ΔTK was produced in SW102 *E. coli* containing pBAC-BoHV-4-A-KanaGalK-ΔTK by heat-induced homologous recombination (Figure 1A). Homologous recombination correctness was demonstrated by HindIII restriction profiling and Southern blotting with a specific probe for CMV promoter sequence (Figure 1C). SW102 containing pBAC-BoHV-4-A-BoSΔRS-HAΔTK were passed 20 times to ensure clonal stability. To obtain BoHV-4-CMV-BoSΔRS-HA-ΔTK infectious viral particles, pBAC-BoHV-4-A-BoSΔRS-HA-ΔTK was electroporated in BEK or BEK*cre* cells, and the later cells depleted the BAC/GFP cassette, as shown by the loss of green plaques (Figure 1D). BoHV-4-A-BoSΔRS-HA-ΔTK replicated similarly to BoHV-4-A parental virus (Figure 1E), and BoHV-4-A-BoSΔRS-HA-ΔTK-infected cells expressed BoSΔRS-HA (Figure 1F–H).

### 2.2. BoHV-4-CMV-BoSΔRS-HA-ΔTK Induces Antibodies That Cross-React with the SARS-CoV-2 Spike Protein

**Figure 2:**
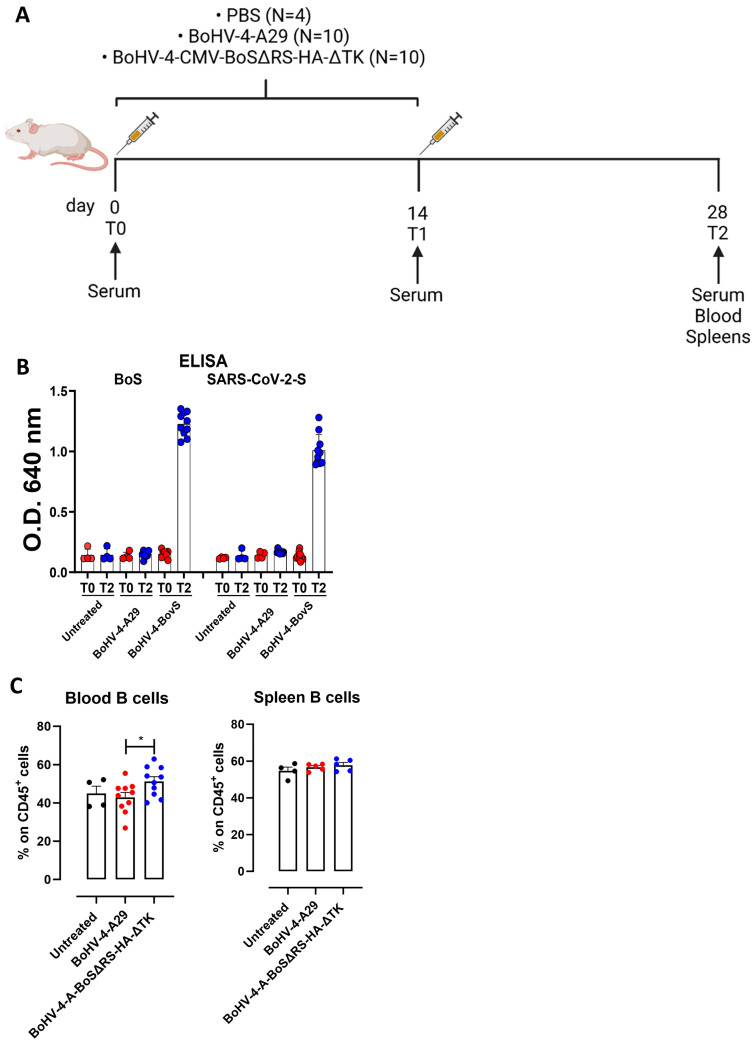
BoHV-4-CMV-BoSΔRS-HA-ΔTK induces antibodies that cross-react with the SARS-CoV-2 spike protein. (**A**) Schematic representation of the vaccination protocol. (**B**) ELISA analysis of the BCoV or SARS-CoV-2 spike-specific antibodies in the sera of mice vaccinated twice with either control BoHV-4-A29 (N = 10) or BoHV-4-CMV-BoSΔRS-HA-ΔTK (N = 10) or left untreated (N = 4), analyzed before starting the treatment (T0), and two weeks after the last immunization (T2). (**C**) FACS analysis of the frequency of CD45^+^ B220^+^ B lymphocytes in the blood and spleens of immunized or control mice, analyzed a T2. Graphs show values from individual mice (N = 10 per immunized groups, N = 4 for untreated mice) and means ± SEM of the percentages of specific lysis. * *p* < 0.05, One-Way ANOVA followed by Tukey’s test.

To assess whether a previous immune response against spike proteins belonging to non-SARS-CoV-2 coronaviruses could induce a cross-reaction against the SARS-CoV-2 spike protein, female BALB/c mice were vaccinated twice with BoHV-4-CMV-BoSΔRS-HA-ΔTK, or with a recombinant BoHV-4 delivering an unrelated expression cassette for a monkeypox virus glycoprotein (BoHV-4-A29) [19] as a control. A further control was established with an untreated group of mice. Immune responses were analyzed two weeks after the second vaccination (Figure 2A).

Vaccination with BoHV-4-CMV-BoSΔRS-HA-ΔTK induced a specific humoral immune response, as high levels of circulating IgG specific for the BoS were present in the sera collected two weeks after the second vaccination (T2) in BoHV-4-CMV-BoSΔRS-HA-ΔTK-vaccinated mice, but not in pre-vaccination sera (T0) nor in sera from untreated control mice. Interestingly, these antibodies cross-reacted with the human coronavirus spike protein (SARS-CoV-2; original Wuhan strain) (Figure 2B), suggesting that immunity to BoS may cross-react with SARS-CoV-2. This humoral response was accompanied by an increase in circulating B lymphocytes in the peripheral blood of mice vaccinated with BoHV-4-CMV-BoSΔRS-HA-ΔTK as compared to BoHV-4-A29-vaccinated or untreated control mice suggesting that BoHV-4-CMV-BoSΔRS-HA-ΔTK induced a clonal expansion of BoS-specific B cells, although no differences in B lymphocytes were observed in the spleens (Figure 2C). The gating strategy used for flow cytometric analysis is shown in Supplementary Figure S1A,B. However, anti-spike-positive sera were unable to neutralize pseudotyped lentiviral vectors with different spike proteins coming from different SARS-CoV-2 variants, thus highlighting the absence or a very low amount under the detection level, of serum neutralizing antibodies.

### 2.3. Immunization with BoHV-4-CMV-BoSΔRS-HA-ΔTK Induces Spike-Specific T Cell Responses

**Figure 3:**
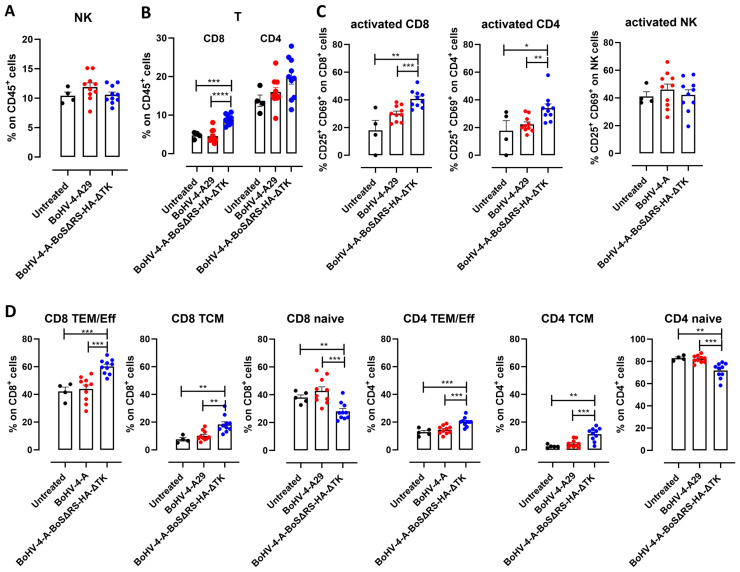
BoHV-4-CMV-BoSΔRS-HA-ΔTK induces T cell expansion and activation. FACS analysis of the frequency of (**A**) CD45^+^ CD3^−^ CD49b^+^ NK cells, (**B**) CD45^+^ CD3^+^ CD8^+^ and CD4^+^ T cells and of the (**C**) activation (CD25^+^ CD69^+^) of T and NK cells and (**D**) of effector/effector memory CD44^+^ CD62L^−^, central memory CD44^+^ CD62L^+^, and naïve CD44^low^ CD62L^+^ CD4^+^ and CD8^+^ T lymphocytes in the peripheral blood of mice vaccinated twice with either control BoHV-4-A29 (N = 10) or BoHV-4-CMV-BoSΔRS-HA-ΔTK (N = 10) or left untreated (N = 4), analyzed two weeks after the last immunization. Graphs show the single mice and the means ± SEM of the frequency of cells. * *p* < 0.05, ** *p* < 0.01, *** *p* < 0.001, **** *p* < 0.0001; One-Way ANOVA followed by Tukey’s test.

Besides a humoral response, immunization with BoHV-4-CMV-BoSΔRS-HA-ΔTK also induced T cell responses. Indeed, two weeks after the second vaccination, no variations in NK cells nor in their activation were observed (Figure 3A,C), but higher frequencies of CD8^+^ and CD4^+^ T lymphocytes were observed in the peripheral blood of BoHV-4-CMV-BoSΔRS-HA-ΔTK-vaccinated mice as compared to the BoHV-4-A29 and the untreated controls (Figure 3B and Supplementary Figure S1A,C). Of note, vaccinated mice displayed increased activation of both CD8^+^ and CD4^+^ T cells (Figure 3C) and expansion of both effector/effector memory CD44^+^ CD62L^−^ and central memory CD44^+^ CD62L^+^ CD4^+^ and CD8^+^ T cells (Figure 3D and Supplementary Figure S1A,D), indicating that BoHV-4-CMV-BoSΔRS-HA-ΔTK induced a specific immune response that was not induced by control BoHV-4-A29. In the spleens, while no alteration in NK cells, CD8^+^, or CD4^+^ T lymphocytes were observed (Supplementary Figure S2A,B), CD8^+^ or CD4^+^ T cell activation and the expansion of effector/effector memory and central memory CD4^+^ and CD8^+^ T cells were induced by BoHV-4-CMV-BoSΔRS-HA-ΔTK vaccination (Supplementary Figure S2C,D).

**Figure 4:**
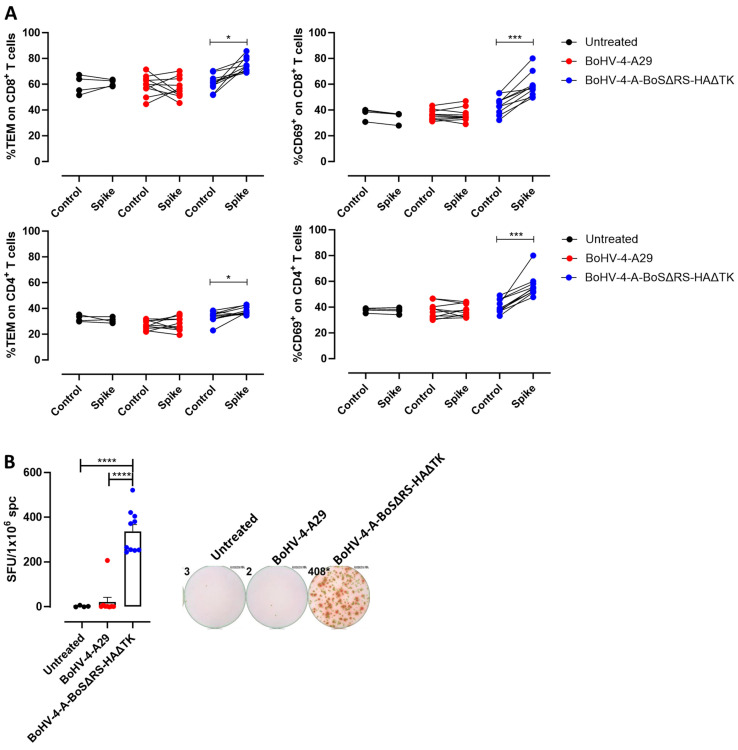
BoHV-4-CMV-BoSΔRS-HA-ΔTK activates spike-specific T cells. (**A**) FACS analysis of the frequency of effector memory CD44^+^ CD62L^−^ and activated CD69^+^ CD8^+^ and CD4^+^ T cells in the spleens of control or vaccinated mice, analyzed after 48 h in vitro re-stimulation with a 1:2 dilution of cell culture supernatants containing or not the bovine coronavirus spike protein. (**B**) IFN-γ ELISpot of splenocytes of control or vaccinated mice, analyzed after 48 h in vitro re-stimulation with a 1:2 dilution of cell culture supernatants containing or not the bovine spike protein. The graph shows the results from single mice and the means ± SEM of number of spot forming units (SFU)/10^6^ cells measured after re-stimulation with spike, calculated after the subtraction of the SFU measured after re-stimulation with the control. Representative images are shown. * *p* < 0.05, *** *p* < 0.001, **** *p* < 0.0001; Student’s *t* test (**A**) and One-Way ANOVA followed by Tukey’s test (**B**).

This T cell response was spike-specific, as in vitro re-stimulation of splenocytes with the BoS induced a statistically significant increase in effector/effector memory and activated CD4^+^ and CD8^+^ T cells in mice vaccinated with BoHV-4-CMV-BoSΔRS-HA-ΔTK, but not in control mice (Figure 4A). Moreover, a high number of IFN-γ-producing cells, as assessed by ELISpot, was induced by in vitro re-stimulation of splenocytes with BoS only in mice vaccinated with BoHV-4-CMV-BoSΔRS-HA-ΔTK, but not in control mice (Figure 4B).

### 2.4. T Lymphocytes Specific for BoS Cross-React with SARS-CoV-2 Spike and Kill Spike-Expressing Cells

**Figure 5:**
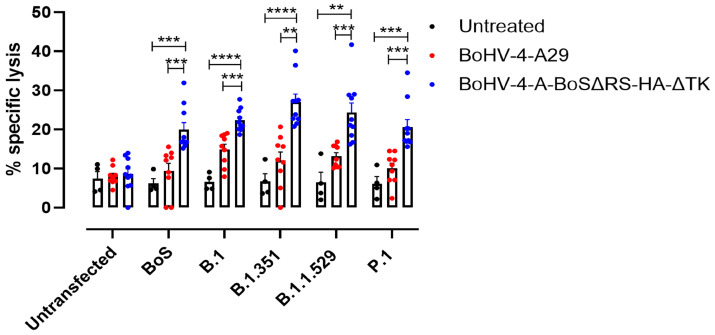
T lymphocytes specific for BoS kill cells expressing SARS-CoV-2 spike. FACS of the percentage of the specific lysis of 3T3-kB cells transfected or not with plasmids coding for spike Wuhan-Hu-1 (B.1 Lineage; China), Beta (B.1.351 Lineage; South Africa), Gamma (P.1 Lineage; Brazil), and Omicron (B.1.1.529 Lineage; Europe) SARS-CoV-2 S or with the pINT2-(TK-CMV-BoSΔRS-HA-TK), or A29, co-cultured for 48 h with splenocytes from untreated mice or mice vaccinated with either control BoHV-4-A29 or BoHV-4-CMV-BoSΔRS-HA-ΔTK. Graphs show values from individual mice (N = 10 per immunized groups, N = 4 for untreated mice) and means ± SEM of the percentages of specific lysis. ** *p* < 0.01, *** *p* < 0.001, **** *p* < 0.0001; One-Way ANOVA followed Tukey’s by test.

To assess the cross-reactivity of the T lymphocytes activated by BoHV-4-A-BoSΔRS-HAΔTK with the SARS-CoV-2 spike and their effector functions, a cytotoxicity experiment was performed by culturing splenocytes from vaccinated or control mice with syngeneic NIH/3T3 mouse fibroblasts stably transfected with the BALB/c major histocompatibility molecule (MHC) H-2Kd and the co-stimulatory molecule B7.1 (3T3/kB) and transiently transfected with plasmids expressing BoSΔRS-HA or SARS-CoV-2 variant spikes. In this system, spike-derived peptides are presented in the context of autologous MHC class I molecules. Interestingly, T cells from BoHV-4-CMV-BoSΔRS-HA-ΔTK-vaccinated mice exerted a cytotoxic activity not only against cells expressing the BoS, but also against cells expressing different variants of SARS-CoV-2 spike. Of note, no significant differences in the amount of cell death induced by splenocytes from BoHV-4-CMV-BoSΔRS-HA-ΔTK-vaccinated mice in cells transfected with BoS or with any of SARS-CoV-2 spike variants was observed, demonstrating that immunization against BoS may induce a protective cellular immunity to the main SARS-CoV-2 variants of concern (Figure 5).

## 3. Discussion

The COVID-19 pandemic was characterized by a wide heterogeneity in the clinical manifestations of SARS-CoV-2-infected subjects, spanning from asymptomatic disease to fatal outcomes. Among the different causes that can be responsible for this, previous infections by other coronaviruses able to induce immune response that cross-react to SARS-CoV-2 antigens has been proposed [1,21,22,23,24]. Indeed, CD4^+^ T cells reacting to the SARS-CoV-2 spike protein have been identified in more than 30% of subjects who had not contracted SARS-CoV-2 infections [5]. Similarly, in vitro re-stimulation with SARS-CoV-2 spike peptides induced CD8^+^ T cell responses in 10–20% of unexposed individuals. About 70% of the peptides recognized by T cells in SARS-CoV-2 naive subjects show high similarity to the seasonal human common cold coronaviruses HCoV-OC43, HCoV-229E, HCoV-HKU1, and HCoV-NL63, suggesting that pre-existing infections by coronaviruses could induce cross-reactive immune responses [25]. In this paper, we have demonstrated that the bovine coronavirus BCoV also induces cross-reactive responses to SARS-CoV-2. Indeed, vaccination of mice with a viral vector encoding BoS induces both humoral and cellular immune responses able to recognize BoS and cross-react with several variants of SARS-CoV-2-S. The cross-reacting antibodies elicited by BoS do not neutralize SARS-CoV-2 infection. This is not a limit to the protective activity exerted by BCoV against SARS-CoV-2, since several studies demonstrated that the presence of pre-infection SARS-CoV-2-reacting antibodies did not protect human subjects from infection, nor was it correlated with disease severity or hospitalization rates [26]. This evidence suggests that a pre-existent humoral response is not required for protective immunity to the virus, which is instead mediated by cellular responses [27,28,29]. In line with this, CD8^+^ T cells specific for BoS are able to cross-react with SARS-CoV-2 spike protein and induce the death of cells expressing several SARS-CoV-S variants of concern.

These findings confirm that exposure to other members of the coronavirus family can contribute to induce protective immune responses against SARS-CoV-2. This may explain why certain individuals were resistant to SARS-CoV-2 infection while others remained asymptomatic upon infection. Of note, our results are particularly interesting, as the BoS protein is highly homologous to HCoV-OC43S and, like HCoV-OC43S, binds to the same receptor, which is, however, a completely different receptor than the SARS-CoV-2 spike. Thus, the characterization of this cross-reaction may contribute to the identification of peptide epitopes conserved among various coronaviruses, providing insights for the development of pan-coronavirus vaccines that can protect from novel SARS-CoV-2 variants of concern and newly arising pandemic coronaviruses.

## 4. Materials and Methods

### 4.1. Cells

Murine NIH/3T3 fibroblasts stably expressing H-2Kd and B7.1 (3T3/kB) were cultured in DMEM (ThermoFisher Scientific, Waltham, MA, USA) supplemented with 20% FBS [19]. Human embryonic kidney (HEK)-293 T cells were purchased from ATCC (CRL-11268, Manassas, VA, USA); Madin–Darby bovine kidney (MDBK; ATCC: CRL-6071), bovine embryo kidney cells (BEK, from the Istituto Zooprofilattico Sperimentale, Brescia, Italy; BS CL-94), and BEK cells expressing *cre* recombinase [30] were grown in complete Eagle’s minimal essential medium (cEMEM, containing 2 mM of L-glutamine, 1 mM of sodium pyruvate, 100 μg/mL of streptomycin, 100 IU/mL of penicillin, and 0.25 μg/mL of amphotericin B (all from Gibco, Waltham, MA, USA), complemented with 10% FBS) at 37 °C with 5% CO_2_ in a humidified incubator.

### 4.2. Plasmids Construction and Transfection in HEK293T Cells

The codon-optimized nucleotide sequence coding for the BCoV spike glycoprotein (BoS), depleted of the last 57 bp, coding for a potential endoplasmic reticulum retrieval signal, and labelled with the hemagglutinin (HA) tag, BoSΔRS-HA, was chemically synthesized (Genescript, Piscataway, NJ, USA) and sub-cloned in a BoHV-4 shuttle vector, pINT2 [18], generating pINT2-(TK-CMV-BoSΔRS-HA-TK). The secreted form of the BCoV and SARS-CoV-2 (original Wuhan variant) spike protein sequences [18,19,31], depleted of the transmembrane domain regions, BoSΔTM-HA and SARS-CoV-2 SΔTM-HA, respectively, were chemically synthesized (Genescript) and sub-cloned in the same shuttle vector, pINT2, obtaining pINT2-TK-CMV-BoSΔTM-HA-TK and pINT2-TK-CMV-SΔTM-HA-TK.

HEK293T cells were seeded into 25 cm2 flasks at a density of 1 × 10^6^ cells per flask and transfected with either pINT2-(TK-CMV-BoSΔRS-HA-TK), pINT2-(TK-CMV-BoSΔTM-HA-TK), pINT2-TK-CMV-SΔTM-HA-TK, or pEGFP-C1 (negative control, Clontech), by means of the polyethyleneimine (PEI) transfection reagent (Polysciences, Inc., Warrington, PA, USA). The transfection procedure was carried out as described in [32]. After transfection, DMEM/F12 medium (1:1 ratio, Euroclone, Pero, Italy) without FBS was added to the cells, which were then incubated for 48 h at 37 °C in a humidified incubator with 5% CO_2_. Supernatants were collected, clarified, and concentrated using Amicon Ultra-100K centrifugal filters (Millipore, Burlington, MA, USA) and subsequently analyzed by immunoblotting to assess protein expression.

### 4.3. Immunoblotting

Protein expression was assessed on the concentrated supernatants, as well as on cell extracts deriving from HEK293T cells transfected with pINT2-(TK-CMV-BoSΔRS-HA-TK) or pEGFP-C1, by Western immunoblotting analysis. BEK cells infected with 0.5 multiplicity of infection (M.O.I.) of BoHV-4-CMV-BoSΔRS-HA-ΔTK or left uninfected were also analyzed by Western blotting as previously described [20]. Membranes were probed with an anti-HA mouse monoclonal antibody (G036, Abm Inc., New York, NY, USA), diluted 1:10,000, followed by addition of horseradish peroxidase-conjugated anti-mouse immunoglobulin (A9044, Merck, Rahway, NJ, USA), diluted 1:15,000. The signals were detected using ChemiDoc (BioRad, Hercules, CA, USA) after the addition of enzymatic substrates (Clarity Max, BioRad).

### 4.4. Bacterial Artificial Chromosome (BAC) Recombineering, Selection, and Southern Blotting Analyses

BAC recombineering was performed in SW102 *E. coli*, containing pBAC-BoHV-4-A-TK-KanaGalK-TK, as described in [18]. The recombineering process involved retargeting the XhoI linearized pINT2-(TK-CMV-BoSΔRS-HA-TK) into BoHV-4 TK locus, containing Kana-GalK selector cassette (https://ncifrederick.cancer.gov/recombineering, accessed on 30 March 2023, to obtain pBoHV-4-CMV-BoSΔRS-HA-ΔTK. The BAC DNA was then analyzed by digestion with the HindIII restriction enzyme and Southern blotting with a specific digoxigenin-labelled probe targeting the CMV promoter. The probe was obtained through PCR employing CMV/BglII-sense 5′-CGA AGA TCT CAT AGC CCA TAT ATG GAG TTC-3′ and CMV/BglII-antisense 5′-GGA AGA TCT CAA AAC AAA CTC CCA TTG ACG-3′ primers [30,32].

### 4.5. Cell Electroporation and Reconstitution, Production, and Titration of the Recombinant Virus

Approximately 5 µg of BAC DNA was electroporated into BEK and BEK*cre* cells in 600 µL DMEM (Euroclone) without serum, using the Bio-Rad Gene pulser Xcell (4 mm gap cuvettes; 270 V, 1500 µF). Once cytopathic effects (CPE) appeared, flasks were frozen, and 3 freezing and thawing cycles were performed to harvest viable viral particles. Permissive cells, such as BEK or MDBK, were employed for viral tittering.

The recombinant BoHV-4s were produced and propagated by infecting permissive BEK or MDBK cells. Viruses were harvested from frozen and thawed infected cells supernatants, and pelleted through a 30% sucrose cushion, as already described [18].

### 4.6. Viral Growth Curves

BEK cells were infected with BoHV-4-A and BoHV-4-A-BoSΔRS-HAΔTK at a M.O.I. of 0.1. Supernatants were collected daily, and the virus was quantified by limiting dilution on permissive cells. Differences in the viral titers measured at the different time points were calculated as the mean of triplicate analysis ± standard errors of the mean (SEM). Statistical significance was determined using Student’s *t*-test (*p* > 0.05 for all time points).

### 4.7. Immunofluorescence Staining and Cytofluorimetric Analyses of BoHV-4-CMV-BoSΔRS-HA-ΔTK Infected Cells

Sub-confluent monolayers of BEK cells were infected with 0.1 M.O.I. of BoHV-4-CMV-BoSΔRS-HA-ΔTK or left uninfected. After 48 h, the cells were fixed using 4% paraformaldehyde and then permeabilized with 0.1% Triton-X 100. Aspecific binding sites were blocked by incubating cells in PBS plus 1% BSA and 10% FBS (Merck) at room temperature for 1 h. Cells were then incubated overnight at 4 °C with a mouse monoclonal anti-HA antibody (1:1000, G036, Abm Inc.), followed by incubation with a goat anti-mouse IgG Alexa 488-conjugated secondary antibody (1:500, A11029, Life Technologies, Carlsbad, CA, USA). Fluorescent images were captured using an inverted fluorescence microscope (Zeiss Axiovert S100, Oberkochen, Germany) equipped with a digital camera (Zeiss Axiocam MRC).

The transduction efficiency of BoHV-4-CMV-BoSΔRS-HA-ΔTK was also quantified by flow cytometry using a FACS Canto II (BD Biosciences, San Jose, CA, USA). Briefly, BEK cells were infected as described above, and 48 h post-infection, detached with trypsin, washed twice in PBS, resuspended in cold PBS, and counted. A total of 10^6^ cells were fixed, permeabilized, and stained as described for the immunofluorescent staining protocol. The percentage of positive cells was measured recording 50000 events per sample, gating on the background signal from non-infected cells or cells incubated with the secondary antibody alone. The data were acquired and analyzed using Diva 9.02 software (BD Bioscience).

### 4.8. Induction of BoV-S-Specific Immune Responses in Mice

Female BALB/c mice were generated and maintained at Molecular Biotechnology Center “Guido Tarone”, University of Torino. The animal studies were performed following the European Directive 2010/63 and approved by the Animal Care and Use Committee of University of Torino and the Italian Ministry of Health. Free access to food and water was guaranteed to mice, which were kept in a 12/12 light/dark cycle. Mice (*n* = 10 per group) were vaccinated twice by intraperitoneal injections of 200 µL of DMEM containing 10^6^ TCID50 (Median Tissue Culture Infectious Dose) of BoHV-4-CMV-BoSΔRS-HA-ΔTK or control BoHV-4-A-A29 (T0). Vaccination was repeated after 2 weeks (T1). Untreated mice were used as a further control (*n* = 4). In total, 24 8-week-old female BALB/c mice were used, randomly allocated into three groups: 10 mice were vaccinated with BoHV-4-CMV-BoSΔRS-HA-ΔTK, coding for BoV-S; 10 mice were vaccinated with a control vaccine, BoHV-4-A-A29; 4 mice were left untreated. The sample size was calculated using G*Power software, version 3.1.9.7, assuming that the humoral response induced by vaccination has an effect size of 0.8, with α = 0.05 and power = 0.95. No inclusion or exclusion criteria were set, and no animals or data were excluded from the study. Single mice represent the experimental units. Within each group, mice were randomly allocated into cages containing 5 animals (4 for untreated mice). Vaccination and retro-orbital blood collection were performed under anesthesia, which was induced by intramuscular injection of Zoletil^®^ and RompunT. To assess the immune response induced by vaccination, two weeks apart from T1 (named T2), mice were anesthetized, blood was collected by cardiac puncture using a 25-gauge needle, followed by cervical dislocation, and then spleens were collected. The spleens were smashed and filtered using a 70 µm pore cell strainer. The resulting cells were pelleted at 1400 rpm for 10 min and incubated in erythrocyte lysing buffer (15.8 mM Na_2_CO_3_, 155 mM NH_4_Cl, and 1 mM EDTA, pH 7.3) for 10 min at room temperature. Cells were then washed in RPMI-1640 supplemented with 10% FBS and used for subsequent experiments. Aliquots of 20 × 10^6^ cells were re-suspended in FBS supplemented with 10% DMSO (Merck) and stored at −80 °C.

### 4.9. Assessment of Spike Cross-Reactive Antibody Levels After Vaccination in Mice by ELISA

Murine sera were tested by an experimenter blind to vaccine treatments for SARS-CoV-2- or BCoV-specific IgG antibodies as described in [33]. Plates were coated with 50 ng/well of HEK-expressing secreted versions of BoS or SARS-CoV-2 (Wuhan variant) spike glycoproteins, which were obtained as described in Section 4.2. All the animal sera were tested diluted 1:10. The secondary anti-mouse IgG conjugated with HRP (A9044, Merck) antibody was used at a 1:15,000 dilution.

### 4.10. Cytotoxicity Assay

3T3/kB cells were cultured in DMEM (ThermoFisher Scientific) with 20% FBS. Cells were transfected with the plasmids expressing four different SARS-CoV-2 spike glycoproteins: Wuhan-Hu-1 (B.1 Lineage; China), Beta (B.1.351 Lineage; South Africa), Gamma (P.1 Lineage; Brazil), and Omicron (B.1.1.529 Lineage; Europe) [20]; alternatively, cells were transfected with the pINT2-(TK-CMV-BoSΔRS-HA-TK) plasmid using Lipofectamine 2000 (following the manufacturer’s instructions). A total of 10^4^ transfected or not transfected 3T3/kB target cells were incubated with 2 µM Carboxyfluorescein succinimidyl ester (CFSE, ThermoFisher Scientific) for 20 min at 37 °C, washed in RPMI-1640 with 10% FBS, and then co-cultured with splenocytes from vaccinated mice for 48 h. Cells were then collected and incubated with 7-Amino-ActinomycinD (7-AAD, 1 μg/mL, BD Biosciences). Samples were acquired on a BD FACSVerse (BD Bioscience) and analyzed using FlowJO10.5.3. Cells were gated on the CFSE^+^ targets, and the percentage of 7-AAD^+^ dead cells was measured. Percent-specific lysis was calculated with the formula [(dead targets in sample (%) − spontaneous dead targets (%))/(dead target maximum-spontaneous dead targets (%))] × 100. Spontaneous dead targets were calculated as the % dead cells measured in target cells cultured in the absence of splenocytes. Maximum dead targets was defined as the percentage of 7-AAD^+^ dead cells measured in target cells treated with PBS supplemented with 1% saponin [19]. Cell killing of non-transfected 3T3/kB target cells was analyzed as a control.

### 4.11. FACS Analysis

An amount of 1 × 10^6^ single cells derived from spleens and heparinized blood, as described in [19], were incubated for 10 min at room temperature with an anti-mouse CD16/CD32 Fc-receptor blocker (ThermoFisher Scientific). Cells were then labeled for 20 min at 4 °C with anti-mouse CD45-VioGreen, CD3-FITC, CD8-VioBlue, CD4-APC-Vio770, B220-PE-Vio770, and CD49b-PE (from Miltenyi Biotec, Bergisch Gladbach, Germany) and CD44-PE, CD25-APC, CD69-PE/Cy7, and CD62L-APC (from Biolegend, San Diego, CA, USA). At least 50,000 CD45^+^ events per sample were acquired using a BD-FACSVerse and analyzed with FlowJO10.5.3. Doublet discrimination was performed, and dead cells were excluded based on 7-AAD staining.

### 4.12. ELISpot

The enzyme-linked immunospot (ELISpot) assay was used to assess spike-specific T-cell responses in mice immunized with BoHV-4-CMV-BoSΔRS-HA-ΔTK. Splenocytes were isolated from vaccinated and control mice as described before and re-suspended in RPMI-1640 plus 10% FBS. Fresh splenocytes were plated at a density of 5 × 10^5^ cells/well in a 96-well ethanol-treated PVDF membrane plate (Millipore, Multiscreen IP) previously coated with anti-IFN-γ capture antibody (BD ELISPOT Mouse IFN-γ Set; Cat: 551083). Splenocytes were stimulated with supernatants containing or not the spike protein, or using Concanavalin A (Merck) 2 µg/mL as a positive control, for 48 h at 37 °C in a 5% CO_2_ incubator. After the incubation, the plates were washed and incubated with a biotinylated anti-mouse IFN-γ detection antibody, followed by Streptavidin-HRP. Spots in the membranes were developed using the 3-ammino-9-ethyl carbazole (AEC) solution and buffer (Merck). To stop the reaction, plates were washed with deionized water. Spots were counted using the CTL ImmunoSpot analyzer (CTL, Shaker Heights, OH, USA), and the results expressed as the number of spot-forming cells (SFCs) per 10^6^ splenocytes, subtracting the spots derived from unstimulated splenocytes.

### 4.13. SARS-CoV Neutralization Assay Against the Original Viral Strain and Variants

Lentiviral vectors pseudotyped with SARS-CoV-2 spike variants were obtained as described before [20]. The pseudoviruses display on their surface different spike glycoprotein variants: Wuhan-Hu-1 (B.1 Lineage; China), Alpha (B.1.1.7. Lineage; United Kingdom), Beta (B.1.351 Lineage; South Africa), Gamma (P.1 Lineage; Brasil), Delta (B.1.617.2 Lineage, India), or Omicron (B.1.1.529 Lineage; Europe) [20].

### 4.14. Statistical Analysis

Statistical analyses were performed by GraphPad Prism (Version 10.0.1, GraphPad Software, Boston, MA, USA). Differences in results from in vitro assays, such as FACS and ELISA data, were analyzed using One-Way ANOVA followed by Tukey’s test or Student’s t test (with Kruskal–Wallis’s correction for samples with different variance). Asterisks indicate significant differences among the groups (* *p* < 0.05, ** *p* < 0.01, *** *p* < 0.001, **** *p* < 0.0001).

## Data Availability

All data generated or analyzed during this study are included in this published article and its supplementary information files.

## Supplementary Materials

The supporting information can be downloaded at: https://www.mdpi.com/article/10.3390/ijms252111509/s1.

## References

[1] Murray S.M., Ansari A.M., Frater J., Klenerman P., Dunachie S., Barnes E., Ogbe A. (2023). The impact of pre-existing cross-reactive immunity on SARS-CoV-2 infection and vaccine responses. Nat. Rev. Immunol..

[2] Grote A., Hiller K., Scheer M., Munch R., Nortemann B., Hempel D.C., Jahn D. (2005). JCat: A novel tool to adapt codon usage of a target gene to its potential expression host. Nucleic Acids Res..

[3] Naqvi A.A.T., Fatima K., Mohammad T., Fatima U., Singh I.K., Singh A., Atif S.M., Hariprasad G., Hasan G.M., Hassan M.I. (2020). Insights into SARS-CoV-2 genome, structure, evolution, pathogenesis and therapies: Structural genomics approach. Biochim. Biophys. Acta Mol. Basis Dis..

[4] Cui J., Li F., Shi Z.L. (2019). Origin and evolution of pathogenic coronaviruses. Nat. Rev. Microbiol..

[5] Braun J., Loyal L., Frentsch M., Wendisch D., Georg P., Kurth F., Hippenstiel S., Dingeldey M., Kruse B., Fauchere F. (2020). SARS-CoV-2-reactive T cells in healthy donors and patients with COVID-19. Nature.

[6] Schulien I., Kemming J., Oberhardt V., Wild K., Seidel L.M., Killmer S., Sagar, Daul F., Salvat Lago M., Decker A. (2021). Characterization of pre-existing and induced SARS-CoV-2-specific CD8(+) T cells. Nat. Med..

[7] Jaago M., Rahni A., Pupina N., Pihlak A., Sadam H., Tuvikene J., Avarlaid A., Planken A., Planken M., Haring L. (2022). Differential patterns of cross-reactive antibody response against SARS-CoV-2 spike protein detected for chronically ill and healthy COVID-19 naive individuals. Sci. Rep..

[8] Vlasova A.N., Saif L.J. (2021). Bovine Coronavirus and the Associated Diseases. Front. Vet. Sci..

[9] Zhu Q., Li B., Sun D. (2022). Advances in Bovine Coronavirus Epidemiology. Viruses.

[10] Zhang X.M., Herbst W., Kousoulas K.G., Storz J. (1994). Biological and genetic characterization of a hemagglutinating coronavirus isolated from a diarrhoeic child. J. Med. Virol..

[11] Han M.G., Cheon D.S., Zhang X., Saif L.J. (2006). Cross-protection against a human enteric coronavirus and a virulent bovine enteric coronavirus in gnotobiotic calves. J. Virol..

[12] Amer H.M. (2018). Bovine-like coronaviruses in domestic and wild ruminants. Anim. Health Res. Rev..

[13] Vijgen L., Keyaerts E., Moes E., Thoelen I., Wollants E., Lemey P., Vandamme A.M., Van Ranst M. (2005). Complete genomic sequence of human coronavirus OC43: Molecular clock analysis suggests a relatively recent zoonotic coronavirus transmission event. J. Virol..

[14] Kin N., Miszczak F., Diancourt L., Caro V., Moutou F., Vabret A., Ar Gouilh M. (2016). Comparative molecular epidemiology of two closely related coronaviruses, bovine coronavirus (BCoV) and human coronavirus OC43 (HCoV-OC43), reveals a different evolutionary pattern. Infect. Genet. Evol..

[15] Szczepanski A., Owczarek K., Bzowska M., Gula K., Drebot I., Ochman M., Maksym B., Rajfur Z., Mitchell J.A., Pyrc K. (2019). Canine Respiratory Coronavirus, Bovine Coronavirus, and Human Coronavirus OC43: Receptors and Attachment Factors. Viruses.

[16] Arenas A., Borge C., Carbonero A., Garcia-Bocanegra I., Cano-Terriza D., Caballero J., Arenas-Montes A. (2021). Bovine Coronavirus Immune Milk Against COVID-19. Front. Immunol..

[17] Tilocca B., Soggiu A., Musella V., Britti D., Sanguinetti M., Urbani A., Roncada P. (2020). Molecular basis of COVID-19 relationships in different species: A one health perspective. Microbes Infect..

[18] Park S.C., Conti L., Franceschi V., Oh B., Yang M.S., Ham G., Di Lorenzo A., Bolli E., Cavallo F., Kim B. (2023). Assessment of BoHV-4-based vector vaccine intranasally administered in a hamster challenge model of lung disease. Front. Immunol..

[19] Conti L., Bolli E., Di Lorenzo A., Franceschi V., Macchi F., Riccardo F., Ruiu R., Russo L., Quaglino E., Donofrio G. (2020). Immunotargeting of the xCT Cystine/Glutamate Antiporter Potentiates the Efficacy of HER2-Targeted Immunotherapies in Breast Cancer. Cancer Immunol. Res..

[20] Donofrio G., Franceschi V., Macchi F., Russo L., Rocci A., Marchica V., Costa F., Giuliani N., Ferrari C., Missale G. (2021). A Simplified SARS-CoV-2 Pseudovirus Neutralization Assay. Vaccines.

[21] Bacher P., Rosati E., Esser D., Martini G.R., Saggau C., Schiminsky E., Dargvainiene J., Schroder I., Wieters I., Khodamoradi Y. (2020). Low-Avidity CD4(+) T Cell Responses to SARS-CoV-2 in Unexposed Individuals and Humans with Severe COVID-19. Immunity.

[22] Le Bert N., Tan A.T., Kunasegaran K., Tham C.Y.L., Hafezi M., Chia A., Chng M.H.Y., Lin M., Tan N., Linster M. (2020). SARS-CoV-2-specific T cell immunity in cases of COVID-19 and SARS, and uninfected controls. Nature.

[23] Grifoni A., Weiskopf D., Ramirez S.I., Mateus J., Dan J.M., Moderbacher C.R., Rawlings S.A., Sutherland A., Premkumar L., Jadi R.S. (2020). Targets of T Cell Responses to SARS-CoV-2 Coronavirus in Humans with COVID-19 Disease and Unexposed Individuals. Cell.

[24] Mahajan S., Kode V., Bhojak K., Karunakaran C., Lee K., Manoharan M., Ramesh A., Hv S., Srivastava A., Sathian R. (2021). Immunodominant T-cell epitopes from the SARS-CoV-2 spike antigen reveal robust pre-existing T-cell immunity in unexposed individuals. Sci. Rep..

[25] Nelde A., Bilich T., Heitmann J.S., Maringer Y., Salih H.R., Roerden M., Lubke M., Bauer J., Rieth J., Wacker M. (2021). SARS-CoV-2-derived peptides define heterologous and COVID-19-induced T cell recognition. Nat. Immunol..

[26] Wang J., Guo C., Cai L., Liao C., Yi H., Li Q., Hu H., Deng Q., Lu Y., Guo Z. (2021). Pre-Existing Cross-Reactive Antibody Responses Do Not Significantly Impact Inactivated COVID-19 Vaccine-Induced Neutralization. Front. Immunol..

[27] Lin C.Y., Wolf J., Brice D.C., Sun Y., Locke M., Cherry S., Castellaw A.H., Wehenkel M., Crawford J.C., Zarnitsyna V.I. (2022). Pre-existing humoral immunity to human common cold coronaviruses negatively impacts the protective SARS-CoV-2 antibody response. Cell Host Microbe.

[28] Anderson E.M., Goodwin E.C., Verma A., Arevalo C.P., Bolton M.J., Weirick M.E., Gouma S., McAllister C.M., Christensen S.R., Weaver J. (2021). Seasonal human coronavirus antibodies are boosted upon SARS-CoV-2 infection but not associated with protection. Cell.

[29] Beretta A., Cranage M., Zipeto D. (2020). Is Cross-Reactive Immunity Triggering COVID-19 Immunopathogenesis?. Front. Immunol..

[30] Donofrio G., Sartori C., Franceschi V., Capocefalo A., Cavirani S., Taddei S., Flammini C.F. (2008). Double immunization strategy with a BoHV-4-vectorialized secreted chimeric peptide BVDV-E2/BoHV-1-gD. Vaccine.

[31] Pratelli A., Capozza P., Minesso S., Lucente M.S., Pellegrini F., Tempesta M., Franceschi V., Buonavoglia C., Donofrio G. (2024). Humoral Immune Response in Immunized Sheep with Bovine Coronavirus Glycoproteins Delivered via an Adenoviral Vector. Pathogens.

[32] Pedrera M., Macchi F., McLean R.K., Franceschi V., Thakur N., Russo L., Medfai L., Todd S., Tchilian E.Z., Audonnet J.C. (2020). Bovine Herpesvirus-4-Vectored Delivery of Nipah Virus Glycoproteins Enhances T Cell Immunogenicity in Pigs. Vaccines.

[33] Amanat F., Stadlbauer D., Strohmeier S., Nguyen T.H.O., Chromikova V., McMahon M., Jiang K., Arunkumar G.A., Jurczyszak D., Polanco J. (2020). A serological assay to detect SARS-CoV-2 seroconversion in humans. Nat. Med..

