# Supplementary material for: Cross-Reactive Immune Response of Bovine Coronavirus Spike Glycoprotein to SARS-CoV-2 Variants of Concern
Source: Int J Mol Sci. 2024 Oct 26;25(21):11509. doi: 10.3390/ijms252111509 (PMC11546235; doi:10.3390/ijms252111509)
Supplement: Supplementary file 1 [file ijms-25-11509-s001.zip › ijms-3203388-supplementary.pdf]

Supplementary Figure 1

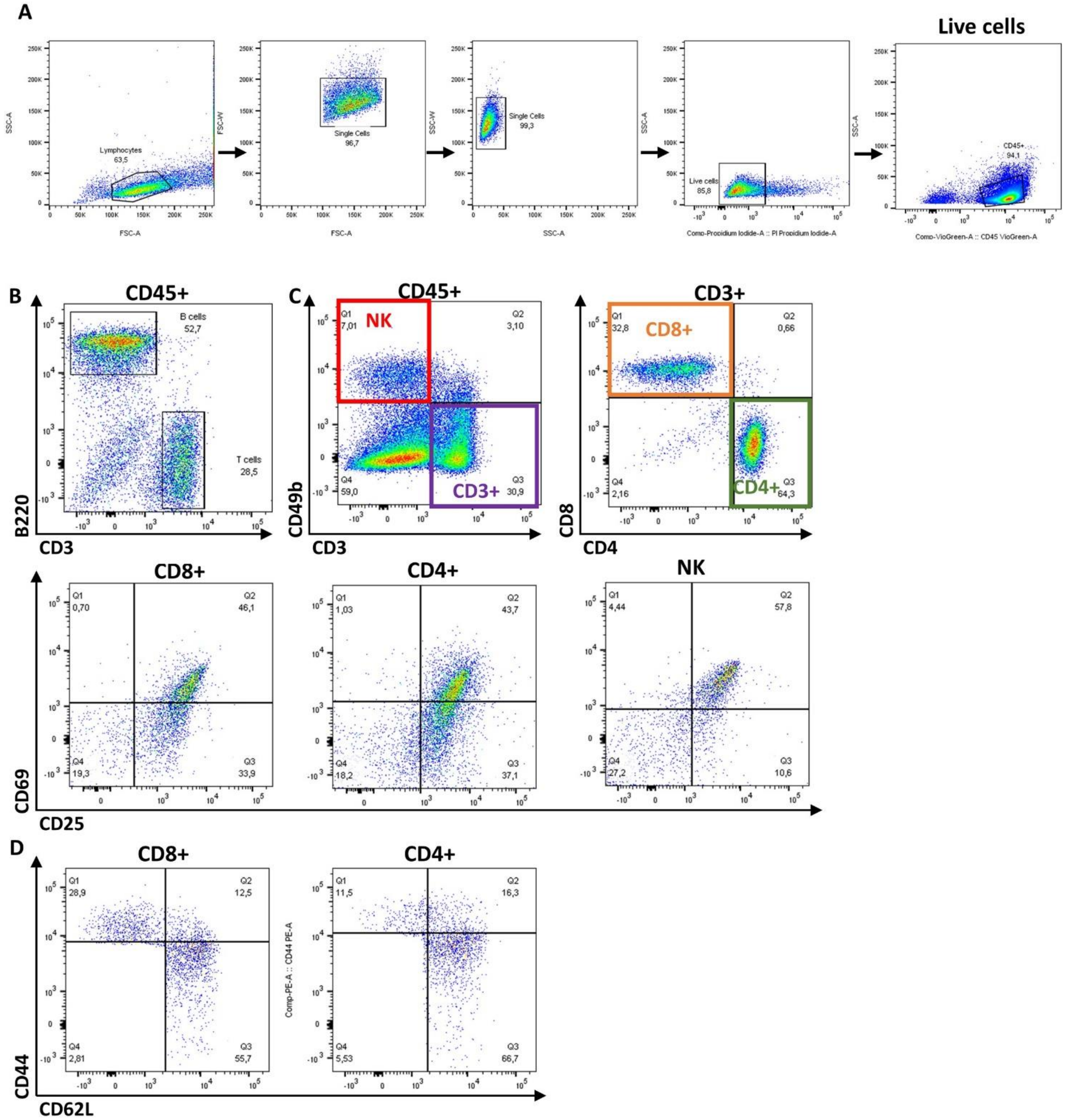

Supplementary Figure 1. Flow cytometric gating strategy. Density plots showing the gating strategy used for the analysis of immune responses. A) In all the analyses, lymphocytes were gated on physical parameters. After doublet exclusion, live cells were selected and gated on CD45+ cells. B) For B cell analysis, B220+ CD3- B lymphocytes were considered. C) T lymphocytes were considered as CD3+ Cd49b- while NK cells as CD3-

CD49b. CD4+ and CD8+ T lymphocytes were gated and C) the activation markers CD25 and CD69 or D) the memory/effector/naïve markers CD44 and CD62L were analyzed.

Supplementary Figure 2

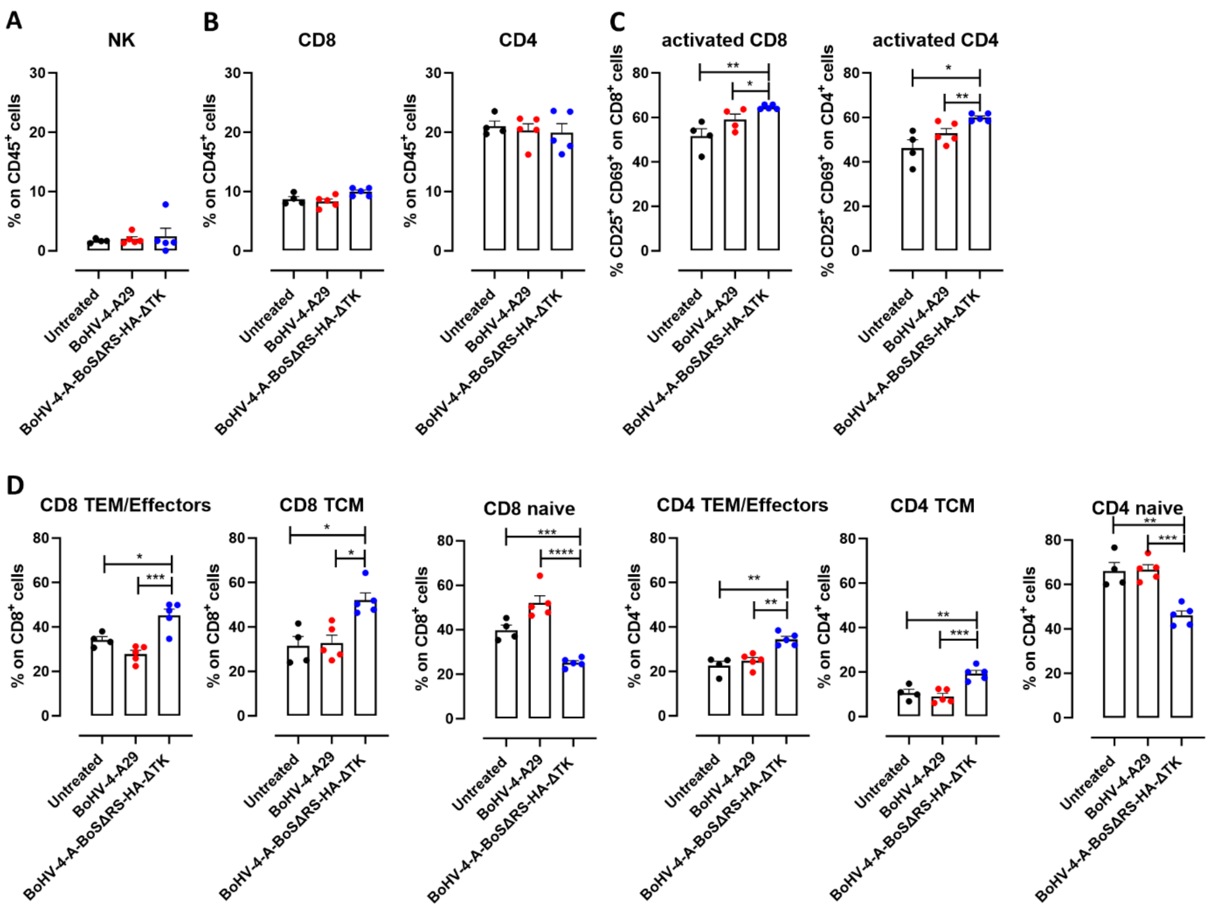

Supplementary Figure 2. BoHV-4-CMV-BoSΔRS-HA-ΔTK induces splenic T cell expansion and activation. FACS analysis of the frequency of A) NK cells, B) $CD4^+$ and $CD8^+$ T cells and C) of the activation and D) memory/effector phenotype of T lymphocytes in the peripheral blood of mice vaccinated twice with either control BoHV-4-A29 (N=5) or BoHV-4-CMV-BoSΔRS-HA-ΔTK (N=5) or left untreated (N=4), analyzed two weeks after the last immunization. Graphs show the single mice and the means ± SEM of the frequency of cells. *P < 0.05, **P < 0.01, ***P < 0.001, ****P<0.0001; and One-Way ANOVA for multiple comparisons.
